# Thai Older Persons' Digital Capacity and Application of Technology for Healthy Aging: A Mixed-Method Study

**DOI:** 10.1155/jare/8080561

**Published:** 2025-11-28

**Authors:** Thet Htoo Pan, Myo Nyein Aung, Saiyud Moolphate, Thin Nyein Nyein Aung, Yuka Koyanagi, Carol Ma Hok Ka, Eun Woo Nam, Jan A. G. M. van Dijk, Motoyuki Yuasa

**Affiliations:** ^1^Department of Global Health Research, Graduate School of Medicine, Juntendo University, Tokyo, Japan; ^2^Faculty of International Liberal Arts, Juntendo University, Tokyo, Japan; ^3^Advanced Research Institute for Health Sciences, Juntendo University, Tokyo, Japan; ^4^Department of Public Health, Faculty of Science and Technology, Chiang Mai Rajabhat University, Chiang Mai, Thailand; ^5^Department of Family Medicine, Faculty of Medicine, Chiang Mai University, Chiang Mai, Thailand; ^6^Global Health and Chronic Conditions Research Group, Chiang Mai University, Chiang Mai, Thailand; ^7^Department of Judo Therapy, Faculty of Health Sciences, Tokyo Ariake University of Medical and Health Sciences, Tokyo, Japan; ^8^S R Nathan School of Human Development, Singapore University of Social Sciences, Singapore; ^9^Department of Health Administration, Software Digital Healthcare Convergence College, Yonsei University, Wonju, Republic of Korea; ^10^Department of Communication Science, University of Twente, Enschede 7500, the Netherlands

**Keywords:** Asia, community, digital inclusion, global health, healthy aging

## Abstract

**Background:**

Thailand has aged fast, becoming a superaging society. Simultaneously, ongoing rapid digital transformation puts older people at risk of being left behind. Research assessing the digital skills of older people and their application of digital technology for health is a literature gap.

**Method:**

This study is an explanatory-sequential design mixed-method study. Community survey of 500 community older adults in Northern Thailand applied London School of Economics and Political Science (LSE) digital skills instrument to measure “operational internet skills,” “information navigation skills,” “social skills,” “creative skills,” and “mobile skills” and perceived health and technology use for health promotion. Quantitatively, Kruskal–Wallis and Mann–Whitney *U* tests identified differences in the digital skills across sociodemographic and internet use characteristics, while multivariable ordinal logistic regressions analyzed types of digital skills associated with health outcomes. Qualitatively, thematic analyses explained the quantitative findings in detail.

**Results:**

The mean age of the participants was 68.36 years, of which 69% were female. Ordinal logistic regression identified that higher “social skills” positively influenced self-rated health (*β* = 0.32, 95% CI = 0.11–0.52), while ‘Information navigation skills' affected internet use to access healthcare (*β* = 0.20, 95% CI = 0.04–0.36). Older adults with higher levels of ‘operational skills' and ‘social skills' positively associated with application of technology for health promotion in terms of improving eating habits (‘operational skills', *β* = 0.54, 95% CI = 0.28–0.79; 'social skills', *β* = 0.64, 95% CI = 0.45–0.83); accessing healthcare (‘operational skills,' *β* = 0.49, 95% CI = 0.23–0.75 ‘social skills' *β* = 0.74, 95% CI = 0.55–0.93) and accessing long-term care services (‘operational internet skills,' *β* = 0.47, 95% CI = 0.21–0.73; ‘social skills' *β* = 0.66, 95% CI = 0.47–0.85). Multiple focus-group interviews of older persons revealed how smart phone apps enabled them to stay connected, seek care and help others.

**Conclusion:**

Therefore, it is urgent to enhance digital skills and internet use among older populations to promote healthy aging.

## 1. Introduction

The population in Thailand has aged very fast and became a superaging society in the early 21^st^ century [1]. In 2024, Thailand has 15.7 million (22.0% of the population) of older adults aged 60 and over, ranking the highest within the Southeast Asian region [2]. 2021–2030 is the UN Decade of Healthy Aging, and Thailand has been investing to promote this [3]. Following the COVID-19 pandemic, digital technology has shown potential in the promotion of healthy aging [4]. For example, Internet use among older people helps to prevent social isolation and dementia [5], promote healthy lifestyles [6], and maintain a strong social network [7–9]. In addition, internet connectivity and digital literacies have become “supersocial determinants of health” because it influences “social determinants of health” such as educational attainment, economic sustainability, and the healthcare system [10]. In health and social care service deliveries, digital technologies overcome physical barriers and limited resources through integrated care and telemedicine [11–13]. Therefore, having access to the internet and sufficient digital skills have become fundamental human rights allowing people to get the full benefits from online health and social services [14].

Internet connectivity among the Thai population is ranked as one of the highest in Asia, yet many older folks are still offline [15]. This situation posts a challenge for many older adults who are being left behind due to digital gaps. This digital gap is defined as the division between people who have access to use of digital media and those who do not [16]. Van Dijk classified the digital gap into three levels: an “access gap,” a “usage gap,” and a “participation gap,” characterized by sequential inequalities [16]. The access gap is narrowing with global efforts to establish universal access to the internet. In 2024, 91.6% of households in Thailand have internet access [17].

Despite Thai people largely having access to the internet, not everyone uses it and some lack the digital skills to do so, known as the second-level digital gap. The second-level digital gap is common among older adults especially in low- and middle-income countries (LMICs) [18]. Only 32.1% of older adults aged 75 and over used the internet, compared to 89.5% of the general population. [15]. Older adult without sufficient digital skills can be left behind in healthy aging as they are not able to participate in health and social activities delivered online [19]. This is known as the third-level digital gap. This digital skills gap and participation gap highlight how urgent it is to promote the internet use and digital skills among older people [20].

The global evidence based on older adults' digital literacy is still limited compared with younger populations. Existing studies on digital literacy were mostly conducted in general population, students, or working groups [21–23]. Earlier research on older adults largely concentrated on technology acceptance, motivational factors [12, 24], or simple binary classifications of Internet use versus nonuse [18]. Since the COVID-19 pandemic, however, scholarly attention to this population has accelerated, though much of the focus has been on digital health literacy or the types of online activities undertaken [22, 25]. Van Dijk proposed a framework on digital skills since 2003, later elaborated by Van Deursen in 2010 [26]. This covered most of the other framework on digital literacies, competencies, and skills conducted in prior research. Moreover, it has been internationally validated by a large representative population. In this study, this framework has been used to measure the digital skills among older adults by applying the “*Digital Skill Measurement Scale*” developed by the LSE [27]. The tool is used for the first time in Thailand context.

Many countries have increasingly recognized the digital gap among older adults, and national policies have begun to prioritize digital literacy interventions [28, 29]. Much of the available evidence, however, comes from small-scale case studies in local contexts [30]. For example, research in Mexico has reported intergenerational training models in which grandchildren support their grandparents in acquiring digital skills [31]. Another example is from Singapore, where peer-learning programs have been started by certified older adults in community centers for older people [32]. While some local authorities in several countries have initiated digital training programs for older adults, these efforts are often not systematically reported. At the same time, stakeholders and supranational organizations continue to debate the most effective models for digital literacy training [33].

Meanwhile, many health and social services have become digitally delivered in Thailand after the COVID-19 pandemic, although the investment for digital inclusion is variable across the country [34]. For example, the Ministry of Public Health in Thailand initiated a five-year plan “eHealth Strategy” aligned with “Thailand 4.0,” the nation's 20-year strategic framework which includes the establishment of eHealth systems [35]. Another scheme set up by the Thai government is the “Digital Wallet.” Ten thousand Thai Baht (approximately US$290) is provided to eligible Thai citizens through a digital wallet to stimulate economic growth [36]. If older adults cannot participate in these online activities due to digital skills gaps, they can be left behind in equitable access to these health services. Indeed, digital skills also encompass foundational aspects of digital health literacy, as higher digital literacy enhances the ability to search, evaluate, and apply online health information to promote healthy lifestyles [37, 38]. Therefore, it is increasingly important to understand how competent older adults are in terms of digital skills and engagement with digital technology for healthy aging.

Research investigating the digital skills among older adults is still very limited in Thailand and Southeast Asia [39, 40]. While recent studies have linked internet use to health outcomes among Thai, older adults they often focus on activity types rather than core digital skills [40]. To assess both skill levels and cultural context, there is a need for evidence based on validated tools combined with qualitative interviews. This study applies a mixed-method approach, to identify digital skills associated with health outcomes and to explain these associations in the cultural context of Thailand.

The current study therefore aimed to (1) measure the internet characteristics and level of digital skills belonging to Thai older adults and (2) assess the association of digital skills with health-related outcomes in four aspects: (i) self-rated health (SRH); (ii) use of the internet and digital technologies to improve eating habits; (iii) use of the internet and digital technologies to access health care; and (iv) use of the internet and digital technologies to access long-term care services. In addition, qualitative inquiry explored how older adults applied internet, applications, and digital technology for promoting health and explain the quantitative results.

## 2. Material and Methods

The study design was mixed-methodology, explanatory-sequential design (Quan > qual) [41]. Quantitatively, we measured the level of 5 digital skills and identified the types of digital skills associated with health outcomes using structured questionnaires. Qualitative interviews followed the survey which helped to explain the results from the quantitative findings. This mixed-method approach provides a more comprehensive understanding of why older people use the internet and how digital skills are applied in the context of healthy aging. This study is part of “Digitally Inclusive, Healthy Aging Communities (DIHAC): A Cross-Cultural Study in Japan, Republic of Korea (ROK), Singapore, and Thailand” [20].

### 2.1. Quantitative Method

The cross-sectional survey recruited community resident older adults from two districts of Chiang Mai province in northern Thailand: Mueang and Hang Dong. The inclusion criteria were (1) older residents aged ≥ 60 years (2) residing in a community which has ongoing community-based health promotion activities. Based on the internet user percentage among older people in Thailand, the sample size of 480 was estimated with a 95% confidence interval and a power of 80%. Data were collected in the community in 2023 by trained research assistants using interviewer-administered questionnaires. A total of 500 participants answered the survey questionnaires.

#### 2.1.1. Research Instruments and Measurements

##### 2.1.1.1. Independent Variables: Digital Skills

The study instruments were transculturally translated from English into Thai versions. The translation followed forward and backward translation according to World Health Organization guidelines, including a pilot study to ensure reliability and validity [42].

The “Digital Skill Measurement Scale” from the LSE measured the digital skills [27]. The twenty-two-item 5-point Likert scale instrument has five domains: “operational internet skills”; “information navigation skills”; “social/communication skills”; “creative skills”; and “mobile internet skills.” Each item ranges from 1 = “Not at all true of me” to 5 = “Very true of me.” The combination of all skills provided a holistic view of a person's ability to function effectively in an online environment. Each digital skill domain ranges from 1 to 5. Higher scores indicate a higher level. All 5 domains of digital skills questionnaire exhibited good internal consistency: “operational internet skills” (*α* = 0.90); “information navigation skills” (*α* = 0.93); “social skills” (*α* = 0.94); “creative skills” (*α* = 0.87); and “mobile internet skills” (*α* = 0.79).

##### 2.1.1.2. Dependent Variables: Health-Related Outcomes

SRH was assessed based on a 4-point Likert scale: 1 = “Very healthy” to 4 = “Not healthy.” This single-item questionnaire measuring general health status showed strong validity and reliability in different populations including older people [43]. The item was reversely coded during the data analysis.

A three-itemed 5-point Likert scale was used to measure the level of digital technology use for health promotion activities. Participants were asked how frequently they use the internet and digital technology to (i) improve eating habits, (ii) access healthcare services, and (iii) access long-term care services. Each item ranged from 0 to 4: 0 = “Never,” 1 = “Rarely,” 2 = “Sometimes,” 3 = “Often,” and 4 = “Usually.”

##### 2.1.1.3. Sociodemographic and Internet Use Characteristics

Sociodemographic characteristics included age, gender, highest educational attainment, current monthly income, and pension status. Two health problems influencing digital device use were included: (i) eye problems and (ii) hand problems when using a mobile phone or a computer [16].

Internet use characteristics comprised mutually nonexclusive data on (i) “Home internet environment type”; (ii) “digital devices used for the internet”; and (iii) “types of social networking services (SNS) used,” with “Yes” or “No” responses. Only the frequency and percentages of “Yes” responses were described in the tables. Digital devices were recoded according to ownership status. The time spent online was measured by asking about the number of hours and days per week spent on the internet. These two were multiplied to obtain the internet usage and then categorized into 4 groups: 0 h = “Nonusers”; < 4 h = “Low users of the internet”; 4 to 24 h = “Regular users”; and ≥ 24 h = “Frequent users” [44].

#### 2.1.2. Data Analysis

Statistical analyses were performed by STATA version 17 SE (Stata Corporation, College Station, TX, USA). Descriptive analysis showed sociodemographic characteristics, internet use characteristics, and the digital skills of community older adults in Thailand. Inferential analyses applied Mann–Whitney *U* test and Kruskal–Wallis's equality-of-populations rank test to detect the differences in digital skills between different sociodemographic and internet use characteristics.

Univariate and multivariable ordinal logistic regression analyses identified association between different types of skills influenced four health-related outcomes: (1) “SRH”; (2) use of the internet and digital technologies to improve eating habits; (3) use of the internet and digital technologies to access health care; and (4) use of the internet and digital technologies to access long-term care services, controlling age, gender, education, and income status in each model. Frequency (percentage) was used to describe categorical data, while mean ± SD was used to describe continuous data. Statistical significance was defined as *p* value < 0.05 with 95% confidence interval. Regression models were checked to be free from multicollinearity.

### 2.2. Qualitative Method

Qualitative interviews were conducted in Chaing Mai Province, Thailand, in 2024. This followed the quantitative phase of the explanatory-sequential design of this mixed-method study. Five focus-group interviews comprising 25 community older adults aged 60 and above with diverse experience in internet use were carried out.

Based on the results from the quantitative phase, participants were interviewed using semistructured questionnaires in order to explain and interpret statistical associations between specific digital skills and health-related outcomes in more detail. Discussions focused on when, why, and how internet and digital technologies were used for active and healthy aging. These questionnaires were designed to identify examples of the five digital skill types measured in the survey (operational, information navigation, social, creative, and mobile skills) and to explore their application in everyday life.

Each focus group lasted between 60 and 120 min. The audio was recorded in verbatim for precise transcription and translation. Bilingual (Thai and English) researchers translated the narratives. Transcriptions were written in English for readability and data familiarization.

The qualitative data were analyzed using MAXQDA software [45, 46]. The coding process was primarily deductive, guided by research questions and constructs emerging from the quantitative phase such as specific digital skills linked to health outcomes, remaining open to inductive coding when new concepts arose from the data, e.g., fear of uploading contents. Analysis began with repeated reading of the translated transcripts, listening to audio recording to achieve deep familiarization [47]. This follows by independent line-by-line semantic coding of a subset of transcripts by two teams of researchers using a preliminary codebook derived from the quantitative findings (e.g., “information navigation skills,” “communication skills,” and “apps use”).

After coding consensus was reached, codes were grouped into broader categories that reflected patterns in digital skill use and health participation, and these categories were reviewed against the full dataset to ensure coherence and distinctiveness. Themes were then clearly defined and named, moving from semantic to latent interpretation, and conceptualized as domain summaries to capture both explicit statements and underlying meanings [47]. Representative quotes were selected to illustrate each theme.

The qualitative findings were integrated into the quantitative survey results, firstly by connecting the sample since some focus-group participants have also answered the survey questionnaires. Secondly, joint display was developed that aligned statistical associations with qualitative themes where the types of digital skills applied were abstracted from the themes that emerged during the coding process [46].

### 2.3. Ethical Approval

The study was approved by the Juntendo University Medical Ethics Committee (approval number E22-0057-M01); date of approval: April 21, 2022; duration of approval for research implementation: April 28, 2022, to March 31, 2026. Written informed consent was obtained from all the participants.

## 3. Results

### 3.1. Quantitative Findings

#### 3.1.1. Sociodemographic Characteristics of the Participants

The mean age of the Thai community older adults in the study was 68.36 ± 6.41 (mean ± SD) years, with most (65.2%) in the young–old group (60–69 years) (Table 1). Female comprised 69.00%. More than half (58.40%) had completed primary school. Approximately half (51.00%) had a monthly income ≥ 3000 Thai Baht, above the national poverty line, although 26.60% had no current income. Five per cent were receiving a pension. Nearly half (48.59%) reported eye problems and 15.23% reported hand problems when using a mobile phone or computer (Table 1).

#### 3.1.2. Digital Skills and Internet Usage Characteristics of Thai Older Adults

Among the five types of digital skills, the Thai older adult participants were most confident in social skills (2.86 ± 1.38), followed by information navigation skills (2.42 ± 1.29), mobile skills (1.86 ± 1.23), operational internet skills (1.74 ± 1.05), and creative skills (1.67 ± 0.94) (Table 2).

Over half (53.00%) used “mobile internet” at home, whilst 26.60% were not using any (Table 2). Most were “smartphone” users (73.80%) whilst 8.60% were still using “analog mobile phones,” PCs (2.40%), or tablets (1.80%). “Single-device” owners accounted for 78.40%, and 17.80% reported no device use (Table 2). On average, older adults in Thailand spent 23.09 ± 31.52 h online per week; 34.0% used the internet more than 24 h per week. The most used SNS was “Line” (64.20%), followed by “YouTube” (55.40%) (Table 2).

#### 3.1.3. Differences Between Digital Skills by Sociodemographic and Internet Usage Characteristics

**Figure 1:**
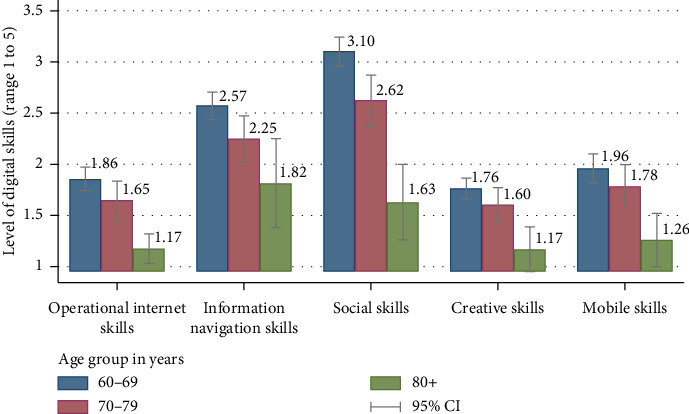
Distribution of five types of digital skills across age group among community older adults in Thailand (*N* = 500).

“Operational internet skills” and “social skills” were significantly higher in women (Supporting Table 1). All five digital skills, except “creative skills” were significantly higher in the 60–69 age group (Figure 1) and in participants with a higher level of education, a higher income status, and who received a pension. Participants with hand problems when using a mobile phone or computer had a lower level of “operational,” “social,” and “creative skills” than those without.

**Figure 2:**
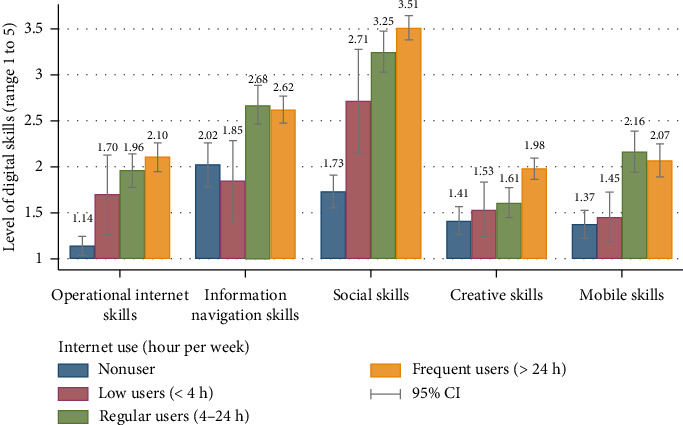
Distribution of five types of digital skills across time spent on internet among community older adults in Thailand (*N* = 500).

The levels of digital skills were significantly different across various internet usage characteristics (Supporting Table 1). “Mobile” and “broadband internet” users, as well as “personal computer” users, were more confident across all skill domains compared to nonusers. Digital skills increased with the number of devices used, with those owning more than 3 devices scored highest in “social skills.” “Frequent internet users” (> 24 h per day) were more confident in “operational”, “social,” and “creative skills,” whilst “Regular users” (4 to 24 h per day) had higher “information navigation” and “mobile skills” (Figure 2). “SNS” users had higher digital skills in all five types than “nonusers” although there were variations according to the types of SNS used. “Facebook messenger” users showed higher “operational,” “information navigation,” “social,” and “mobile” skills (Supporting Table 1).

#### 3.1.4. Health-Related Outcome Distribution: SRH and Digital Technology Use for Health Promotion

Most participants rated their health as “very healthy” (23.65%) or “moderately healthy” (69.14%) (Table 3). However, not all individuals used the internet or digital technology for health promotion activities. One-third (33.80%) had never used the internet and digital technology to improve eating habits. Only 6.84% reported “usually” engaged in this behavior. Similarly, 34.20% had “never” used these technologies to access healthcare. For long-term care services, 43% reported “never” using the internet, with only 6.20% reported “usually” doing so (Table 3).

Subsequent inferential analysis sought to discover how different types of digital skills would impact specific health outcomes.

#### 3.1.5. Digital Skills and Application of Technology for Health

Univariate analysis revealed that each type of digital skills had a positive association with each health-related outcome, except for “creative skills” in relation to SRH (Table 4). Several types of digital skills remained significant in the multivariable ordinal logistic regression model after controlling age, gender, education, and income status. “Social skills” were positively associated with SRH of the older adults (*β* = 0.32, 95% CI = 0.11–0.52, *p* value < 0.01), while “creative skills” showed an inverse relationship (*β* = −0.38, 95% CI = −0.66–−0.10, *p* value < 0.01) (Table 4).

There were variations amongst the types of digital skills influencing three outcome models regarding internet and digital technology use for health promotion activities. Usage of the internet and digital technology to improve eating habits was associated with “operational internet skills” (*β* = 0.54, 95% CI = 0.28–0.79, *p* value < 0.001); “social skills” (*β* = 0.64, 95% CI = 0.45–0.83, *p* value < 0.001); and “creative skills” (*β* = −0.28, 95% CI = −0.52–−0.03, *p* value < 0.05) (Table 5).

Similarly, usage of the internet and digital technology to access healthcare services was influenced by “operational internet skills” (*β* = 0.49, 95% CI = 0.23–0.75, *p* value < 0.001), “social skills” (*β* = 0.74, 95% CI = 0.55–0.93, *p* value < 0.001), and “creative skills” (*β* = −0.35, 95% CI = −0.59–−0.10, *p* value < 0.01), as well as with “information navigation skills” (*β* = 0.20, 95% CI = 0.04–0.36, *p* value < 0.05) (Table 6).

Moreover, positive associations were also found between “operational internet skills” (*β* = 0.47, 95% CI = 0.21–0.73, *p* value < 0.001) and “social skills” (*β* = 0.66, 95% CI = 0.47–0.85, *p* value < 0.001), and the usage of the internet and digital technologies to access long-term care services after sociodemographic variables is controlled (Table 7).

The following section presents qualitative results that provide a more detailed explanation of the quantitative associations in the context of Thailand.

### 3.2. Qualitative Findings

The following themes emerged from the thematic analysis: (1) health-related activities; (2) hobby and daily activities; and (3) social connection (Table 8). Each theme provides examples of how measured digital skills are applied to promote healthy aging.

#### 3.2.1. Health-Related Activities

Quantitative analysis indicated that “social skills” are linked to better health. “Information navigation skills” are linked to using the internet to access healthcare services and that higher “operational skills” lead to health promotion activities. This theme shows how older adults practice those skills in Thailand. Many used Google to search for health-related information, from relieving symptoms and improve eating habits, to access information from trusted sources such as hospitals and government agencies. LINE groups provided regular health updates, while YouTube was used for exercise routines. Thai language-based apps, such as “Mor Phrom” (translated as “Doctor is ready”) developed by the Thai Ministry of Public Health, were used during the COVID-19 pandemic for vaccination verification. The app continues to share information on emerging diseases. The “CalTracker” app helped some older adults monitor calorie intake, including for traditional Thai foods and chain restaurants. Moreover, some of the participants themselves were caregivers for their spouse. They used LINE to coordinate with health volunteers.

These activities drew on “information navigation skills” (searching and evaluating online health information), “social skills” (communicating with service providers), and “operational internet/mobile skills” (using smartphones and apps), mirroring the skill domains linked to positive health outcomes in the survey.

*“I joined the Ramadhipadi Hospital and Chulalongkorn Hospital LINE group. Then they send health information messages to me.”*

*“I looked for the information about the medication I am taking. what are the side effects. Is that good or not. My mobile phone is my library.”*

*“Even small things can be searched. For example, you can look up whether a bug bite is dangerous. I mostly use Google.”*

*“I followed YouTube exercise.”*

*“Everyone uses ‘Mor Phrom' app. This was famous in COVID to check the history of vaccine. Even now, after COVID, the app continues to give information related to diseases such as Dengue and Diabetes. Now we get Monkey Pox information.”*

*“I was diagnosed with piles. I searched the Internet and found that I should avoid certain foods. I should not eat spicy food. I followed that and it really changed. Now I don't have to take any medicine.”*

*“I subscribed to an app called ‘CalTracker'. I checked the calories for the food I eat.”*

#### 3.2.2. Hobbies and Daily Activities

The survey showed that “operational,” “information navigation,” and “social skills” were higher among frequent internet users and those with multiple devices. This theme uncovers how those skills were applied beyond health contexts. Most participants mentioned using Google, YouTube, and Facebook for their hobbies such as cooking and entertainment. Religious studies using the internet were mentioned by one female participant living in this predominantly Buddhist country setting. One participant used her phone to learn the Lana language; others used, “Google maps” for navigation and a Thai language–based app for weather information. Facebook was a medium for sharing activities and generating income through small-scale online sales.

*“I didn't know how to cook. I searched on ‘Google' and ‘YouTube'. Now I can do very well. Cooking is my expertise. Let me show you the picture.”*

*“I use my phone for religion. I study religion. I'm interested in the Lana language. My smartphone can help me learn to write about Lana.”*

*“It's amazing how old songs can still be found on Google.”*

*“I sell things on Facebook. I do it with my family. And I posted it on Facebook. Every day after we get the order, I go to deliver.”*

#### 3.2.3. Social Connection

Quantitative results showed that “social skills” and “operational skills” were positively associated with all health outcomes. This theme discloses how participants used those skills to maintain relationships. “LINE” was predominantly used for chatting, sending, and receiving messages individually or via group chats. Participants applied “social skills” and “operational skills” to sustain these connections.

However, many expressed reluctances to use “creative skills” (e.g., producing and uploading videos or content on “TikTok” or “YouTube”) due to fear of critical comments or a perception that such platforms were for younger people.

*“I don't like TikTok. It's for the young generation to create and post.”*

*“TikTok is only for YouTuber to get more likes. If you get more likes, you get more income. For us, we don't need income from YouTube.”*

*“You get negative comments if the content you upload is not good.”*

## 4. Discussion

The population of Thailand, an aged society and a middle-income economy, is at risk of “growing old before it grows rich”. Meanwhile, it is becoming more and more digitalized, especially in the context of health and social care delivery. Therefore, promoting healthy aging through digital inclusion is timely and necessary to address the challenges of this demographic transition. Grounded on the digital divide theory, this mixed-method study identified five types of digital skills belonging to the Thai older adults and examined their association with various health outcomes. Qualitatively we identified the pathways for using the internet towards healthy aging in the context of Thailand.

Among the five types of digital skills investigated, the study participants were most confident about their “social skills” (Table 2). This suggests that older adults are likely to be socially active online. Of the study sample, 68.00% used “SNS,” with a high frequency being LINE (64.20%) (Table 2). This finding is similar to that from other East Asian contexts, such as the ROK, where culturally embedded messaging platforms “KakaoTalk” enhances older adults' online communications with “social skills” scoring the highest [6]. On the other hand, in European contexts, “operational skills” were more developed [23, 27]. The variations reveal that digital divide needs to be understood in the context of local digital technologies such as apps used, language, and cultural practices. In addition, the COVID-19 pandemic might have led older adults' “social skills” to be higher than other domains due to social distancing and the adoption of online communication apps.

The findings of the current study are consistent with previous literature on how digital skills relate to inequalities between sociodemographic groups. Young–old group (60–69 years) had higher levels of digital skills in all five types compared to those aged 80 years and older, and women scored higher in “operational skills” and “mobile skills” (Supporting Table 1 and Figure 1). Qualitative data revealed that some female participants were village health volunteers, a role that requires digital communication via the “Smart AorSorMor” app to coordinate with other volunteers, government agencies, and care recipients [48]. In Thailand, the role of village health volunteers, predominantly older women, reveals how community-based engagement can enhance digital capacity [49]. In recent study among Thai older internet users, older women were increasing using the internet that no gender gap was observed [40].

Conversely, other social determinants of health were superimposed on digital skills gaps. Among participants, 63.60% had only primary school education or below, and their digital skills were significantly lower than those of university graduates (Supporting Table 1). Similarly, lower income was consistently associated with lower digital skills across all domains. Notably, 26.60% of participants reported having no income, while only 5% were pensioners (Table 1). In addition, certain age-related health problems further worsen the digital gap. “Operational,” “social,” and “creative skills” were significantly lower among those with hand problems in using mobile phones or computers (Supporting Table 1), a condition present in 15% of the sample (Table 1). Our study concurs with previous research emphasizing that socioeconomically disadvantaged groups experience a digital gap and tend to use digital technology less [6, 25, 50]. Since the country's health and social care services are transitioning to be online, the digital gap can exacerbate the existing social inequalities [51]. These intersecting inequities should be addressed in digital inclusion among older adults.

Our study identified the broader LMIC patterns in device use and connectivity. More participants used “mobile internet” than “broadband internet” at home (53.00% vs 42.20%), and smartphones were the dominant device (73.80%), while PC use was rare (2.40%) (Table 2). PC users, however, reported higher confidence in all types of digital skills, and multidevice users tended to have higher skills overall (Supporting Table 1). Among the participants in our sample, 26.60% did not have a home internet environment, yet ITU statistics showed household access to the internet was 91.6% nationwide [52]. Our study sample includes older adults aged 60 and over, which may explain this lower percentage of internet use in the home environment. Mobile phones and the mobile internet are more widely used in LMICs compared to high-income countries (HICs) where computers and broadband are more prevalent [53]. This implies that promoting access and usage of the internet based on the mobile internet and smartphones may lead to a better adoption rate and sustained internet use particularly within limited infrastructure contexts such as in LMICs. In addition, country-level analyses have demonstrated that internet usage is a key socioecological factor positively associated with healthy life expectancy and the probability of becoming a centenarian [54, 55]. Therefore, it has never been more urgent to bring the remaining 26.6% of older adults online to promote healthy aging.

This study also quantified internet usage. On average, Thai older adults spend 23.09 h online per week (Table 2). Higher time spent online leads to greater confidence in “operational,” “socials,” and “creative skills” (Supporting Table 1 and Figure 2). While there is an ongoing debate on whether extended internet use is beneficial, evidence suggests the effect may differ by age group [5, 8, 9]. In the qualitative findings, Thai older adults described using the internet for wide range of purposes, including maintaining social connection, accessing health information and services, and engaging in leisure activities through platforms such as YouTube and Google and Thai language–based apps (Table 8). These insights suggest that, for older adults, greater time online may reflect purposeful and health-supportive engagement rather than passive or harmful use.

Our study examined clear association between specific type of digital skills and health consequences, with “social skills” emerging as a particular important domain. In the quantitative analysis, higher “social skills” had a positive impact on SRH (*β* = 0.32, 95% CI = 0.11–0.52, *p* value < 0.01) (Table 4). Thai older adults tend to be socially active online, enjoying various activities which make them perceive they are healthy. “Social skills” are increasingly essential for online social connection and exchanging information [16]. The application of “social skills” in daily life by older adults was explained in detail by the qualitative findings. In Thailand, “LINE” was the mostly frequently mentioned platform, used for both personal and for group communication. On the other hand, like many countries, domestic migration of younger family members, especially from rural to urban areas, such online communication is vital in order for people to sustain intergenerational and social inclusion utilizing “social skills.” Similar patterns have been reported in the ROK where older adults with higher “social skills” are more likely to be healthy [6]. Not only should digital literacy training programs empower older adults to be socially connected online but also a focus on strengthening “social skills” will lead to better health through social inclusion and access to health and social services.

“Operational internet skills” also played a key role. Together, “operational” and “social skills” were positively associated with internet and digital technology use related to three health promotion activities: (1) to improve eating habits (Table 5); (2) to access healthcare (Table 6); and (3) to access long-term care services (Table 7). The qualitative findings revealed how “operational skills” supported digital device use such as saving online information. On the other hand, “social skills” enabled the sharing and discussion of that information within personal networks. The qualitative findings revealed that the study participants received and shared health information via LINE group chat. Moreover, long-term care services were communicated via LINE from village health volunteers (Table 8). “Information navigation skills” are key to the utilization of digitally delivered health information and care services (Tables 6 and 8). Nowadays where information is widely accessible online, these skills enable older adults to navigate websites and identify accurate, trustworthy sources. In our study, participants with higher information navigation were more likely to use digital technology use to access healthcare (Table 6), often applying them to active searches via “Google” for diet improvements or symptoms management (Table 8). Although the older adults in our study showed a certain level of “information navigation skills” (2.42 out of 5), this still needs to be promoted at the population level to enable people to function more effectively online (Table 2). Therefore, it is essential to include an “information navigation skills” module in digital literacy improvement programs.

Digital skills are integral to the interests, hobbies, and needs of older adults, indicating their value to healthy aging. Of the five types of digital skills, “creative skills” were the most challenging for the study participants, ranking the lowest (Table 2). “Creative skills” relate to the ability to create content and disseminate it publicly online. The results of the qualitative interviews indicated that the participants were apprehensive regarding potential negative feedback when sharing their own content on online platforms such as YouTube and TikTok. This helps to explain the reverse association between “creative skills” and health-related outcomes since older adults perceived that “creative skills” were not essential for them, in fact, making them feel fearful and anxious. This suggests that not all digital skill domains are equally valued or beneficial for older adults, and that training should prioritize those most relevant to health engagement.

In this study, older adults' digital skills were assessed quantitatively, while the qualitative component revealed how participants applied these skills to search, evaluate, and use online health information to support healthy lifestyles (Tables 2, 4, 5, 6, 7, and 8). Importantly, digital skills intersect with social determinants of health (Supporting Table S1). Thus, digital skills function as a foundation for broader digital health literacy competencies that ultimately influence health behaviors and outcomes. These digital skills are increasingly essential for older adults to participate in health promotion interventions, particularly as emerging technologies such as artificial intelligence (AI), Internet of Things (IoT), and immersive platforms become more integrated into public health programs. For example, one AI- and IoT-based healthcare program conducts monitoring online, such as assessing dietary practices and health information monitoring [56]. Moreover, a recent study on immersive virtual reality (IVR) experiences based on natural environments found that they significantly improved stress relief and happiness among older adults [57]. In this context, digital skills have become fundamental to enabling older adults to utilize AI and IoT integrated health promotion programs. Therefore, continuous learning opportunities to sustain and update digital skills are required towards inclusive future for aging society.

Although we identified how older adults use the internet for a healthy lifestyle, over a third of the study participants “never” used the internet and digital technology for health promotion activities (Table 3). The participation gap (3^rd^ degree digital gap) is still significant. This finding concurs with findings from another Thai study and highlights the risk of exclusion as health and care services are digitalized rapidly [40].

Recently, policies to promote healthy aging and digital inclusion have been enacted and implemented across Thailand. The phase-3 National Action Plan for Older Persons and the 2024 Integrated Plan for Aging Society seek to enhance the well-being of older adults including improving digital literacy through schools for older adults and older people associations [28, 58]. Local municipalities are also involved in promoting healthy aging in collaboration with universities. For example, in the study district in Chiang Mai province, a new model of universities for older people includes digital literacy training in the local communities [59]. Meanwhile, Thailand is undergoing rapid digitalization, such as with the Ministry of Public Health's “eHealth Strategy” and “Digital wallet” schemes [35, 36]. Delivery of integrated social services is increasingly transforming to be online, for example, through social media apps such as LINE.

The current levels of digital skills among the older adults in our study are encouraging. However, a large digital gap remains. One in three of the study participants did not use any social media apps nor spend any time on the internet, whilst the gap is wider for participation in health promotion activities (Tables 2 and 3). Therefore, it is imperative to leverage older adults with sufficient digital skills to enable them to get the full benefits from essential services, in so doing leading to healthy aging. Moreover, it is important to include socioeconomic disadvantaged individuals, such as the older age groups, those with lower education and lower income, and those with physical problems using digital devices when implementing digital literacy training programs. It is hoped that our findings will guide policies and programs to strategize digital literary training among older adults.

### 4.1. Strengths and Limitations

The current study is new within the context of Thailand as well as existing global literature. Different types of digital skills were measured applying internationally validated instruments including “operational skills,” “information navigation skills,” “social skills,” “creative skills,” and “mobile skills,” leading to robust regression models. The study provided a deeper insight into the remaining gray digital gap and examined the ways to bring about digital inclusion within a community-based context in the LMIC setting. In addition, the mixed-methodology design enabled us to uncover the specific details regarding digital inclusion of older adults. Qualitative narratives explained the quantitative findings in a Quan > qual approach. The current study is not without limitations. First, the cross-sectional nature of the study limits the interpretation of the associations in terms of causal inference. Future longitudinal or experimental research will confirm temporal and causal relationships. Second, this study recruited community-dwelling older adults, who are likely to be healthier individuals. As our aim is to inform digital literacy training in community settings, future research should investigate digital skills among older adults in different contexts, such as those living in institutions or with poorer health status. Finally, since digital skills are dynamic, future studies may demand multiple measurements applying sophisticated techniques. Despite the limitations, it is hoped that digital literacy training programs will benefit from the results of this study to boost the digital skills among older adults.

### 4.2. Implications

Empowering older adults with digital inclusion requires contextually sensitive strategies. The levels of digital skills among the Thai older people in our study revealed encouraging findings. However, there is still a large digital gap across all levels. In terms of the usage gap, our study showed that 18% of older adults did not use any digital devices whilst 26% did not have a home internet environment. As Thailand is still investing in its infrastructure to enhance internet access, the mobile internet might be more easily accessible than broadband internet for older adults with limited resources. Family members may play an important role in encouraging older adults to use the internet by supporting the adoption of digital devices.

Communities are expected to enhance the digital inclusion of older adults, especially those living alone, notably in rural areas where the internal migration of younger generations to urban areas is increasing resulting in shrinking family sizes. Policies and programs should invest in community digital literacy training, especially for those in older age groups, and with lower levels of education and income. Our study has clearly identified the types of digital skills required to promote healthy aging. Enhancing “operational internet skills,” “information navigation skills,” and “social skills” promotes health through participation in healthy lifestyle modifications. Existing health promotion activities can be integrated within digital technologies, such as creating chat groups in social apps enhancing social connectivity among older adults. At the same time, it is important to consider who the trainers will be in those community digital literacy training programs.

In the context of Thailand, the role of village health volunteers has gained significant attention by delivering essential social services where long-term care systems are lacking. The volunteers mainly use social communication apps such as LINE to connect with care recipients. By promoting digital skills of older adults, these services become accessible. They are also one of the community resources that can help older adults improve their digital skills. Equally, community digital training programs can promote the digital skills of active healthy older adults allowing them to become village health volunteers when their digital skills are competent enough to receive online training from the Ministry of Public Health in order to deliver those services. Other examples include intergenerational programs with high school or university students as extracurricular activities and peer group learning opportunities. Since policies and programs to empower older adults are rising in Thailand, community resources should be mobilized to promote digital inclusion. Apart from encouraging health-related internet use, digital literacy training programs should focus on what older adults value. Our study found that older adults use the internet for hobbies such as watching YouTube videos, learning new languages, singing, and cooking. Digital training programs are recommended to also include tailored sessions geared to what the older adults want to learn.

At the macrolevel, our findings can serve as a guide to the digital literacy training programs in schools for older persons established by the Ministry of Social Development and Human Security, Community Universities for Older People, and countries with similar contexts to strategize the empowering digital inclusion of older adults.

## 5. Conclusion

Thailand is experiencing rapid population aging necessitating the development of integrated care models. The integration of digital technologies holds considerable promise in promoting healthy aging. If we ignore the digital inclusion of older adults, they will be excluded from society and become less independent. The digital gap still remains in Thailand, with 30% of older people offline in terms of the usage gap and 43% offline in terms of the participation gap. Our study has clearly described what types of digital skills are necessary to be promoted. “Social skills,” “operational internet skills,” and “information navigation skills” positively influence health outcomes through apps such as LINE, YouTube, and Google in terms of social connection and access to health and care services to transition to healthier lifestyles. Policies and programs aiming to promote digital skills among older adults are urgent for creating digitally inclusive healthy aging communities.

## Figures and Tables

**Table 1:** Sociodemographic characteristics of Thai community older adults in the survey (*N* = 500).

Variable		Frequency (percentage)	Mean ± SD
Gender	Male	155 (31.00)	
	Female	345 (69.00)	

Age (years)	Young–old (60–69 years)	326 (65.20)	
	Old–old (70–79 years)	133 (26.60)	
	Oldest–old (aged 80 and older)	41 (8.20)	
			68.36 ± 6.41

Education status	Did not go to school	26 (5.20)	
	Primary school graduate	292 (58.40)	
	Junior high school graduate	77 (15.40)	
	High school graduate	77 (15.40)	
	University graduate or higher	28 (5.60)	

Income	No current income	133 (26.60)	
	< 3000 Thai Baht	112 (22.40)	
	≥ 3000 Thai Baht	255 (51.00)	

Pension	Yes	25 (5.00)	
	No	475 (95.00)	

Eye problem when using a mobile phone or a computer	Yes	242 (48.59)	
	No	256 (51.41)	

Hand problems when using a mobile phone or a computer (e.g., Flexion deformity)	Yes	76 (15.23)	
	No	423 (84.77)	

**Table 2:** Digital skills and internet usage characteristics of Thai community older adults in the survey (*N* = 500).

Variable		Frequency (percentage)	Mean ± SD
Digital skills (range 1 to 5)	Operational internet skills		1.74 ± 1.05
	Information navigation skills		2.42 ± 1.29
	Social skills		2.86 ± 1.38
	Creative skills		1.67 ± 0.94
	Mobile skills		1.86 ± 1.23

Types of internet environment at home^∗^	Nonuser	133 (26.60)	
	Mobile internet	265 (53.00)	
	Broadband (fiber, ADSL)	211 (42.20)	
	Do not know	14 (2.80)	

Types of digital devices use^∗^	Nonuser	89 (17.80)	
	Smartphone	369 (73.80)	
	Mobile phone	43 (8.60)	
	Personal computer (PC)	12 (2.40)	
	Tablet	9 (1.80)	

Digital device ownership	Nonuser	89 (17.80)	
	Single device owner	392 (78.40)	
	2 devices owner	16 (3.20)	
	≥ 3 devices owner	3 (0.60)	

Time spent on internet (hours/week)	Nonuser (0)	152 (30.40)	
	Low user of the internet (< 4)	21 (4.20)	
	Regular user (4 to 24)	157 (31.40)	
	Frequent user (> 24)	170 (34.00)	
			23.09 ± 31.52

Types of SNS use^∗^	Nonuser	160 (32.00)	
	Line	321 (64.20)	
	YouTube	277 (55.40)	
	Facebook	102 (20.40)	
	Facebook Messenger	54 (10.80)	
	TikTok	24 (4.83)	
	Others	17 (3.40)	

^∗^Types of internet environment at home, types of digital devices use, and types of SNS use are not mutually exclusive. Only the number and percentage of answering “Yes” were reported.

**Table 3:** Distribution of outcomes: self-rated health and participation in health promotion activities among community resident older adults in Thailand (*N* = 500).

Variable		Frequency (percentage)
Self-rated health	Very healthy	118 (23.65)
	Moderately healthy	345 (69.14)
	Not very healthy	34 (6.81)
	Not healthy	2 (0.40)

*Participation in health promotion activities*		
Usage of the internet and digital technology to improve eating habits	Never	168 (33.80)
	Rarely	98 (19.72)
	Sometimes	122 (24.55)
	Often	75 (15.09)
	Usually	34 (6.84)

Usage of the internet and digital technology to access healthcare	Never	171 (34.20)
	Rarely	106 (21.20)
	Sometimes	94 (18.80)
	Often	87 (17.40)
	Usually	42 (8.40)

Usage of the internet and digital technology to access long-term care services (e.g., house assistance, house cleaning, home bathing, and others)	Never	215 (43.00)
	Rarely	117 (23.40)
	Sometimes	76 (15.20)
	Often	61 (12.20)
	Usually	31 (6.20)

**Table 4:** Association of digital skills with self-rated health among community resident older adults in Thailand.

Variable		Univariate analysis			Multivariable analysis		
		*β*	95% CI	*p* value	*β*	95% CI	*p* value
Digital skills	Operational skills	0.44	0.26–0.62	< 0.001	0.25	−0.03–0.53	0.081
	Information navigation skills	0.20	0.05–0.34	0.008	0.05	−0.11–0.22	0.536
	Social skills	0.38	0.23–0.53	< 0.001	0.32	0.11–0.52	0.002
	Creative skills	0.00	−0.20–0.21	0.985	−0.38	−0.66–−0.10	0.007
	Mobile skills	0.25	0.10–0.41	0.001	0.10	−0.14–0.35	0.403

Income	No current income	0 (ref)			0 (ref)		
	< 3000 Thai Baht	0.26	−0.30–0.81	0.361	0.35	−0.22–0.93	0.230
	≥ 3000 Thai Baht	0.34	−0.11–0.80	0.141	0.14	−0.35–0.63	0.578

Education	Did not go to school	0 (ref)			0 (ref)		
	Primary school graduate	0.83	−0.05–1.72	0.066	0.65	−0.27–1.58	0.163
	Junior high school graduate	0.73	−0.24–1.71	0.139	0.52	−0.50–1.54	0.318
	High school graduate	1.06	0.08–2.04	0.034	0.55	−0.49–1.58	0.299
	University graduate and higher	1.69	0.57–2.82	0.003	0.69	−0.57–1.94	0.284

Age		0.00	−0.03–0.03	0.981	0.02	−0.01–0.06	0.143

Gender	Male	0 (ref)			0 (ref)		
	Female	0.20	−0.21–0.61	0.344	0.00	−0.44–0.45	0.988

*Note:* Self-rated health was measured by 4-point Likert scale ranging from “Not very healthy” to “Very healthy”; significant was defined as *p* value< 0.05; *β* = regression coefficient, ordinal logistic regression.

**Table 5:** Association of digital skills with internet and digital technology use for health promotion activities to improve eating habits among community resident older adults in Thailand.

Variable		Univariate analysis			Multivariable analysis		
		*β*	95% CI	*p* value	*β*	95% CI	*p* value
Digital skills	Operational skills	0.96	0.77–1.14	< 0.001	0.54	0.28–0.79	< 0.001
	Information navigation skills	0.43	0.29–0.56	< 0.001	0.15	−0.01–0.30	0.063
	Social skills	0.90	0.75–1.04	< 0.001	0.64	0.45–0.83	< 0.001
	Creative skills	0.32	0.14–0.50	0.001	−0.28	−0.52–−0.03	0.029
	Mobile skills	0.44	0.30–0.58	< 0.001	−0.19	−0.41–0.02	0.082

Income	No current income	0 (ref)			0 (ref)		
	< 3000 Thai Baht	−0.15	−0.62–0.32	0.537	−0.30	−0.81–0.21	0.254
	≥ 3000 Thai Baht	0.72	0.33–1.12	< 0.001	0.03	−0.40–0.46	0.890

Education	Did not go to school	0 (ref)			0 (ref)		
	Primary school graduate	1.24	0.40–2.08	0.004	0.99	0.04–1.93	0.041
	Junior high school graduate	2.07	1.17–2.97	< 0.001	1.85	0.85–2.86	< 0.001
	High school graduate	2.71	1.80–3.62	< 0.001	1.96	0.94–2.97	< 0.001
	University graduate and higher	2.79	1.73–3.84	< 0.001	1.11	−0.11–2.33	0.073

Age		−0.09	−0.12–−0.07	< 0.001	−0.04	−0.07–−0.01	0.013

Gender	Male	0 (ref)			0 (ref)		
	Female	0.83	0.49–1.18	< 0.001	0.63	0.24–1.03	0.002

*Note:* Digital technology use for health promotion activities to improve eating habits was measured by 5-point Likert scale ranging from 0 to 4, Never to Usually; *β* = regression coefficient, ordinal logistic regression.

**Table 6:** Association of digital skills with internet and digital technology use for health promotion activities to access healthcare services among community resident older adults in Thailand.

Variable		Univariate analysis			Multivariable analysis		
		*β*	95% CI	*p* value	*β*	95% CI	*p* value
Digital skills	Operational skills	1.05	0.86–1.24	< 0.001	0.49	0.23–0.75	< 0.001
	Information navigation skills	0.51	0.37–0.65	< 0.001	0.20	0.04–0.36	0.012
	Social skills	1.03	0.88–1.18	< 0.001	0.74	0.55–0.93	< 0.001
	Creative skills	0.32	0.15–0.50	< 0.001	−0.35	−0.59–−0.10	0.005
	Mobile skills	0.54	0.40–0.68	< 0.001	−0.08	−0.30–0.14	0.463

Income	No current income	0 (ref)			0 (ref)		
	< 3000 Thai Baht	−0.27	−0.74–0.20	0.262	−0.38	−0.91–0.14	0.147
	≥ 3000 Thai Baht	0.69	0.30–1.08	0.001	−0.04	−0.47–0.39	0.850

Education	Did not go to school	0 (ref)			0 (ref)		
	Primary school graduate	0.99	0.17–1.80	0.017	0.69	−0.24–1.63	0.147
	Junior high school graduate	1.72	0.85–2.59	< 0.001	1.34	0.34–2.33	0.009
	High school graduate	2.49	1.60–3.37	< 0.001	1.60	0.59–2.62	0.002
	University graduate and higher	2.99	1.93–4.04	< 0.001	0.92	−0.29–2.14	0.136

Age		−0.11	−0.13–−0.08	< 0.001	−0.05	−0.08–−0.02	0.002

Gender	Male	0 (ref)			0 (ref)		
	Female	0.77	0.42–1.12	< 0.001	0.50	0.10–0.90	0.014

*Note:* Digital technology use for health promotion activities to access healthcare services was measured by 5-point Likert scale ranging from 0 to 4, Never to Usually; significant was defined as *p* value < 0.05; *β* = regression coefficient, ordinal logistic regression.

**Table 7:** Association of digital skills with internet and digital technology use for health promotion activities to access long-term care services among community resident older adults in Thailand.

Variable		Univariate analysis			Multivariable analysis		
		*β*	95% CI	*p* value	*β*	95% CI	*p* value
Digital skills	Operational skills	0.91	0.73–1.09	< 0.001	0.47	0.21–0.73	< 0.001
	Information navigation skills	0.41	0.28–0.55	< 0.001	0.14	−0.03–0.30	0.097
	Social skills	0.90	0.75–1.06	< 0.001	0.66	0.47–0.85	< 0.001
	Creative skills	0.29	0.11–0.48	0.002	−0.21	−0.46–0.03	0.090
	Mobile skills	0.45	0.31–0.59	< 0.001	−0.18	−0.41–0.04	0.106

Income	No current income	0 (ref)			0 (ref)		
	< 3000 Thai Baht	−0.35	−0.83–0.13	0.147	−0.53	−1.05–0.00	0.049
	≥ 3000 Thai Baht	0.46	0.07–0.85	0.020	−0.18	−0.62–0.26	0.419

Education	Did not go to school	0 (ref)			0 (ref)		
	Primary school graduate	0.82	−0.02–1.66	0.057	0.56	−0.40–1.53	0.253
	Junior high school graduate	1.62	0.72–2.52	< 0.001	1.26	0.25–2.28	0.014
	High school graduate	2.03	1.12–2.94	< 0.001	1.28	0.25–2.31	0.015
	University graduate and higher	2.80	1.72–3.88	< 0.001	1.14	−0.09–2.37	0.068

Age		−0.09	−0.12–−0.06	< 0.001	−0.05	−0.08–−0.01	0.006

Gender	Male	0 (ref)			0 (ref)		
	Female	0.62	0.26–0.97	0.001	0.36	−0.05–0.77	0.081

*Note:* Digital technology use for health promotion activities to access long-term care services was measured by 5-point Likert scale ranging from 0 to 4, never to usually; significant was defined as *p* value < 0.05; *β* = regression coefficient, ordinal logistic regression.

**Table 8:** A joint display of qualitative themes explaining how and why Thai community older adults use the internet and digital technologies to promote healthy aging with integration of digital skills in quantitative study.

Theme	Subtheme	Media/apps used	Digital skills applied/integration with quantitative results
Health-related activities	Access to information Relieve symptoms Improve eating habits Access to services Physical activity	LINE group Institutions Village health volunteers Google YouTube Health apps MorPhrom Calorie tracker	Information navigation skills Operational skills Social skills

Hobbies and daily activities	Cooking Religion Entertainment Korean movies Songs Navigation Weather forecast Economic productivity	Netflix Google map Facebook YouTube Thai language-based rain app	Information skills Operational skills Social skills

Social connection	SNS: Individual Group chat Text messages Call	LINE Facebook messenger	Social skills Operational skills

*Note:* Three themes explain why older adults use the internet to promote healthy aging in their daily lives. The subthemes reveal these in more detail. “Media/apps used” reveals the ways in which older adults use the internet. These activities are linked to the digital skills applied to explain the quantitative findings.

## Data Availability

The data presented in this study are available on request from the corresponding author due to privacy reasons.

## Supporting Information

Supporting Table 1 contains distribution of digital skills across sociodemographic characteristics and internet usage characteristics among community resident older adults in Thailand (*N* = 500). Mann–Whitney *U* test and Kruskal–Wallis's equality-of-populations rank test detected the differences in digital skills.
