# Supplementary material for: Thai Older Persons' Digital Capacity and Application of Technology for Healthy Aging: A Mixed-Method Study
Source: J Aging Res. 2025 Nov 28;2025:8080561. doi: 10.1155/jare/8080561 (PMC12680461; doi:10.1155/jare/8080561)
Supplement: Supporting Information — Additional supporting information can be found online in the Supporting Information section. [file 8080561.f1.docx]

**Supplementary Table 1** Distribution of Digital skills across socio-demographic characteristics and internet usage characteristics among community resident older adults in Thailand (N = 500)

| Variable |  | Frequency | Types of Digital Skills (Mean ± SD) | | | | | | | | | |
| --- | --- | --- | --- | --- | --- | --- | --- | --- | --- | --- | --- | --- |
|  |  | (Percentage) | Operational internet skills | | Information Navigation skills | | Social skills | | Creative skills | | Mobile skills | |
| Mann-Whitney U test/ Kruskal–Wallis’s equality-of-populations rank test | | |  | p-value |  | p-value |  | p-value |  | p-value |  | p-value |
| Overall digital skills (Range 1 to 5) |  |  | 1.74 ±1.05 | | 2.42 ±1.29 |  | 2.86 ±1.38 |  | 1.67 ±0.94 |  | 1.86 ±1.23 |  |
| Gender | Male | 155 (31.00) | 1.59 ±1.03 | 0.0021 | 2.41 ±1.35 | 0.6575 | 2.48 ±1.40 | <0.0001 | 1.64 ±0.96 | 0.3403 | 1.88 ±1.27 | 0.7128 |
|  | Female | 345 (69.00) | 1.81 ±1.05 |  | 2.43 ±1.27 |  | 3.03 ±1.34 |  | 1.69 ±0.94 |  | 1.85 ±1.21 |  |
| Age (Years) | Young-old (60–69 years) | 326 (65.20) | 1.85 ±1.05 | 0.0001 | 2.57 ±1.24 | 0.0001 | 3.11 ±1.27 | 0.0001 | 1.76 ±0.93 | 0.0001 | 1.97 ±1.25 | 0.0001 |
|  | Old-old (70–79 years) | 133 (26.60) | 1.65 ±1.11 |  | 2.25 ±1.33 |  | 2.62 ±1.47 |  | 1.60 ±0.98 |  | 1.78 ±1.23 |  |
|  | Oldest-old (aged 80and older) | 41 (8.20) | 1.17 ±0.46 |  | 1.82 ±1.39 |  | 1.63 ±1.18 |  | 1.18 ±0.69 |  | 1.26 ±0.83 |  |
| Education status | Didn’t go to school | 26 (5.20) | 1.29 ±0.82 | 0.0001 | 1.91 ±1.19 | 0.0001 | 2.14 ±1.36 | 0.0001 | 1.61 ±1.01 | 0.0001 | 1.54 ±1.02 | 0.0001 |
|  | Primary School Graduate | 292 (58.40) | 1.51 ±0.91 |  | 2.30 ±1.38 |  | 2.58 ±1.40 |  | 1.49 ±0.90 |  | 1.57 ±1.05 |  |
|  | Junior High School Graduate | 77 (15.40) | 1.79 ±0.90 |  | 2.35 ±0.97 |  | 3.04 ±1.10 |  | 1.98 ±0.85 |  | 2.05 ±1.06 |  |
|  | High School Graduate | 77 (15.40) | 2.09 ±1.04 |  | 2.75 ±1.08 |  | 3.46 ±1.17 |  | 1.86 ±0.98 |  | 2.32 ±1.41 |  |
|  | University Graduate or higher | 28 (5.60) | 3.49 ±1.06 |  | 3.49 ±1.09 |  | 4.21 ±0.84 |  | 2.21 ±1.01 |  | 3.38 ±1.48 |  |
| Income | No current income | 133 (26.60) | 1.48 ±0.94 | 0.0001 | 2.35 ±1.44 | 0.0244 | 2.52 ±1.49 | 0.0001 | 1.48 ±0.87 | 0.0001 | 1.65 ±1.19 | 0.0001 |
|  | <3000 Thai Baht | 112 (22.40) | 1.68 ±1.13 |  | 2.33 ±1.37 |  | 2.55 ±1.38 |  | 1.74 ±1.08 |  | 1.71 ±1.22 |  |
|  | >=3000 Thai Baht | 255 (51.00) | 1.90 ±1.04 |  | 2.50 ±1.17 |  | 3.17 ±1.25 |  | 1.75 ±0.91 |  | 2.04 ±1.23 |  |
| Pension | Yes | 25 (5.00) | 2.84 ±1.34 | <0.0001 | 3.21 ±1.31 | 0.0021 | 3.64 ±1.56 | 0.0020 | 2.14 ±1.36 | 0.0773 | 2.94 ±1.62 | 0.0003 |
|  | No | 475 (95.00) | 1.68 ±1.00 |  | 2.38 ±1.28 |  | 2.82 ±1.36 |  | 1.65 ±0.91 |  | 1.80 ±1.18 |  |
| Difficulty in eyesight in using a mobile phone or a computer | Yes | 242 (48.59) | 1.76 ±1.06 | 0.8096 | 2.49 ±1.33 | 0.3657 | 2.86 ±1.43 | 0.9066 | 1.65 ±0.97 | 0.1830 | 1.88 ±1.26 | 0.9793 |
|  | No | 256 (51.41) | 1.73 ±1.04 |  | 2.35 ±1.24 |  | 2.88 ±1.33 |  | 1.70 ±0.92 |  | 1.85 ±1.21 |  |
| Hand problems using a mobile phone or a computer? (Eg: Flexion deformity) | Yes | 76 (15.23) | 1.58 ±1.01 | 0.0138 | 2.56 ±1.51 | 0.7763 | 2.23 ±1.41 | <0.0001 | 1.59 ±1.05 | 0.0223 | 1.74 ±1.21 | 0.1458 |
|  | No | 423 (84.77) | 1.77 ±1.06 |  | 2.40 ±1.25 |  | 2.97 ±1.35 |  | 1.69 ±0.93 |  | 1.88 ±1.24 |  |
| Internet usage characteristics | | | | | | | | | | | | |
| Types of Internet environment at home* | Non-user | 133 (26.60) | 1.15 ±0.66 | <0.0001 | 2.04 ±1.49 | <0.0001 | 1.75 ±1.13 | <0.0001 | 1.43 ±0.97 | <0.0001 | 1.38 ±0.98 | <0.0001 |
|  | Mobile internet | 265 (53.00) | 2.05 ±1.09 | <0.0001 | 2.61 ±1.12 | <0.0001 | 3.50 ±1.12 | <0.0001 | 1.79 ±0.87 | <0.0001 | 2.15 ±1.29 | <0.0001 |
|  | Broadband (Fibre, ADSL) | 211 (42.20) | 2.01 ±1.12 | <0.0001 | 2.59 ±1.17 | 0.0006 | 3.29 ±1.17 | <0.0001 | 1.81 ±0.95 | 0.0001 | 2.13 ±1.33 | <0.0001 |
|  | Do not know | 14 (2.80) | 1.00 ±0.00 | 0.0001 | 1.81 ±0.64 | 0.0006 | 1.83 ±0.55 | <0.0001 | 1.87 ±0.76 | 0.1817 | 1.75 ±0.58 | 0.3204 |
| Types of digital devices use* | Non-user | 89 (17.80) | 1.15 ±0.73 | <0.0001 | 2.22 ±1.72 | 0.0001 | 1.69 ±1.24 | <0.0001 | 1.38 ±1.07 | <0.0001 | 1.38 ±1.11 | <0.0001 |
|  | Smartphone | 369 (73.80) | 2.55 ±1.75 | <0.0001 | 2.76 ±1.43 | <0.0001 | 3.35 ±1.61 | <0.0001 | 1.46 ±0.59 | <0.0001 | 2.51 ±1.51 | <0.0001 |
|  | Mobile phone | 43 (8.60) | 1.70 ±1.02 | <0.0001 | 1.55 ±0.87 | 0.0006 | 2.03 ±1.15 | <0.0001 | 1.43 ±0.63 | 0.0029 | 1.44 ±0.62 | <0.0001 |
|  | Personal computer | 12 (2.40) | 4.27 ±0.7 | <0.0001 | 3.33 ±0.5 | <0.0001 | 3.80 ±0.72 | <0.0001 | 2.00 ±0.2 | 0.0154 | 2.33 ±1.15 | <0.0001 |
|  | Tablet | 9 (1.80) | 3.62 ±1.54 | 0.0005 | 3.63 ±1.04 | 0.1298 | 4.07 ±1.23 | 0.0034 | 1.73 ±0.61 | 0.3889 | 3.31 ±1.38 | 0.0049 |
| Digital device ownership | Non-user | 89 (17.80) | 1.15 ±0.73 | 0.0001 | 2.22 ±1.72 | 0.0001 | 1.69 ±1.24 | 0.0001 | 1.38 ±1.07 | 0.0001 | 1.38 ±1.11 | 0.0001 |
|  | Single device owner | 392 (78.40) | 1.81 ±1.00 |  | 2.43 ±1.16 |  | 3.06 ±1.26 |  | 1.71 ±0.88 |  | 1.89 ±1.18 |  |
|  | 2 devices owner | 16 (3.20) | 2.96 ±1.51 |  | 3.03 ±1.16 |  | 3.99 ±1.20 |  | 2.04 ±0.98 |  | 3.13 ±1.53 |  |
|  | ≥3 devices owner | 3 (0.60) | 4.47 ±0.92 |  | 4.60 ±0.53 |  | 5.00 ±0.00 |  | 3.53 ±1.62 |  | 4.67 ±0.58 |  |
| Time spent on internet (hours per week) | Non-user (0) | 152 (30.40) | 1.14 ±0.65 | 0.0001 | 2.02 ±1.48 | 0.0001 | 1.73 ±1.10 | 0.0001 | 1.41 ±0.95 | 0.0001 | 1.37 ±0.96 | 0.0001 |
|  | Low users of the Internet (<4) | 21 (4.20) | 1.70 ±1.01 |  | 1.85 ±1.02 |  | 2.71 ±1.32 |  | 1.53 ±0.70 |  | 1.45 ±0.63 |  |
|  | Regular users (4 to 24) | 157 (31.40) | 1.94 ±1.14 |  | 2.68 ±1.32 |  | 3.26 ±1.41 |  | 1.62 ±1.04 |  | 2.17 ±1.41 |  |
|  | Frequent users (>24) | 170 (34.00) | 2.10 ±1.03 |  | 2.62 ±0.98 |  | 3.51 ±0.88 |  | 1.98 ±0.78 |  | 2.07 ±1.18 |  |
| Types of SNS use* | Non-user | 160 (32.00) | 1.15 ±0.66 | <0.0001 | 1.98 ±1.45 | <0.0001 | 1.76 ±1.13 | <0.0001 | 1.44 ±0.98 | <0.0001 | 1.39 ±0.99 | <0.0001 |
|  | Line | 321 (64.20) | 2.07 ±1.09 | <0.0001 | 2.60 ±1.13 | <0.0001 | 3.42 ±1.14 | <0.0001 | 1.82 ±0.92 | <0.0001 | 2.13 ±1.28 | <0.0001 |
|  | YouTube | 277 (55.40) | 2.04 ±1.07 | <0.0001 | 2.69 ±1.12 | <0.0001 | 3.45 ±1.12 | <0.0001 | 1.81 ±0.91 | <0.0001 | 2.10 ±1.25 | <0.0001 |
|  | Facebook | 102 (20.40) | 2.46 ±1.11 | <0.0001 | 2.87 ±1.14 | <0.0001 | 3.81 ±0.89 | <0.0001 | 1.89 ±0.81 | <0.0001 | 2.38 ±1.34 | <0.0001 |
|  | Facebook Messenger | 54 (10.80) | 2.58 ±1.07 | <0.0001 | 2.93 ±1.19 | 0.0007 | 3.82 ±0.88 | <0.0001 | 1.78 ±0.79 | 0.0213 | 2.43 ±1.34 | 0.0006 |
|  | TikTok | 24 (4.83) | 2.23 ±1.40 | 0.1081 | 3.05 ±1.63 | 0.1046 | 3.50 ±1.62 | 0.0147 | 1.58 ±1.06 | 0.1517 | 2.46 ±1.62 | 0.1106 |
|  | Others | 17 (3.40) | 2.68 ±1.41 | 0.0007 | 3.13 ±1.25 | 0.0220 | 3.35 ±1.28 | 0.1005 | 2.67 ±1.45 | 0.0015 | 2.97 ±1.59 | 0.0030 |

Note: * Types of internet environment at home, Types of digital devices use, and Types of SNS use are not mutually exclusive. Only the number and percentage of answering “Yes” were reported.
